# Prognostic Influence of Preoperative Mammographic Breast Density in Operable Invasive Female Breast Cancer

**DOI:** 10.1038/s41598-018-34297-8

**Published:** 2018-10-30

**Authors:** Ki-Tae Hwang, A. Jung Chu, Jongjin Kim, Jong Yoon Lee, Ji Hyun Chang, Sohee Oh, Young A. Kim, Jiwoong Jung, Bumjo Oh

**Affiliations:** 1 Department of Surgery, Seoul Metropolitan Government Seoul National University Boramae Medical Center, Seoul, Republic of Korea; 2 Department of Radiology, Seoul Metropolitan Government Seoul National University Boramae Medical Center, Seoul, Republic of Korea; 3 Department of Radiation Oncology, Seoul Metropolitan Government Seoul National University Boramae Medical Center, Seoul, Republic of Korea; 4 Department of Biostatistics, Seoul Metropolitan Government Seoul National University Boramae Medical Center, Seoul, Republic of Korea; 5 Department of Pathology, Seoul Metropolitan Government Seoul National University Boramae Medical Center, Seoul, Republic of Korea; 6 Department of Surgery, Seoul Medical Center, Seoul, Republic of Korea; 7 Department of Family Medicine, Seoul Metropolitan Government Seoul National University Boramae Medical Center, Seoul, Republic of Korea

## Abstract

We aimed to investigate the potential of preoperative mammographic breast density (MBD) as a prognostic factor in breast cancer. Data of 969 patients with primary breast cancer were analyzed. We defined low MBD as fatty or fibroglandular breast, and high MBD as heterogeneously dense or extremely dense breast, respectively. The high MBD group demonstrated a superior overall survival rate compared to the low MBD group (p < 0.001). Favorable prognostic effects of high MBD were observed in subgroups aged >50 years (p < 0.001) and with positive hormone receptor (HRc) and negative human epidermal growth factor receptor 2 (HER2) (p < 0.001). The high MBD group had a higher proportion of patients aged ≤50 years (p < 0.001) and patients with body mass index (BMI) ≤25 kg/m^2^ (p < 0.001), and a higher proportion of patients who received chemotherapy (p < 0.001). MBD was a significant independent prognostic factor by multivariable analysis (hazard ratio, 0.382; 95% confidence interval, 0.206–0.708). The high MBD group was associated with superior overall survival rates. Preoperative MBD was a strong independent prognostic factor in operable primary invasive female breast cancer, especially in patients with age >50 years and the HRc(+)/HER2(−) subtype. Favorable clinicopathologic features, active treatments, and other factors could contribute to this causality.

## Introduction

Mammographic breast density (MBD) depends on the relative composition of radio-dense fibroglandular tissue relative to radiolucent fat tissue in the breast. In 1976, Wolfe first proposed a four category-based classification system of MBD^1^ and reported the risk of MBD in breast cancer development^2^. Currently, Breast Imaging-Reporting and Data System (BI-RADS) proposed by the American College of Radiology recommends a succinct description of overall breast composition^3^.

Several aspects of the clinical significance of MBD in breast cancer have been investigated. First, the association between MBD and breast cancer development has been extensively studied, and MBD has been reported to be one of the strongest risk factors associated with breast cancer^4,5^. Risk of breast cancer development was reported to be four to six times greater in women with dense breasts compared to women with fatty breasts^6,7^. Second, the question of whether reductions in MBD, (noted after tamoxifen treatment of estrogen receptor-positive breast cancer), are associated with breast cancer prognosis, has been investigated. Both premenopausal^8^ and postmenopausal patients^9^ with estrogen receptor-positive breast cancer, who experience a large reduction in MBD following initiation of tamoxifen treatment, have been reported to have a better prognosis. Third, the association between MBD and breast cancer prognosis has also been investigated, but the results have not been consistent and overall, remain controversial. Some papers reported that fatty breast was associated with worse prognosis^10,11^, but others reported completely opposite results suggesting a favorable prognostic effect of fatty breast^12,13^. Still other studies reported no association between MBD and breast cancer prognosis^14–16^.

As previous studies have focused on the role of MBD as an etiologic factor rather than a prognostic factor in breast cancer, the prognostic value of MBD remains largely unknown. In this study, we aimed to investigate the role of preoperative MBD as a prognostic factor in breast cancer using our institution’s breast cancer database.

## Methods

### Patients

The subjects of this study were consecutive, operable, primary invasive female breast cancer patients, who underwent curative surgery at the Seoul Metropolitan Government Seoul National University Boramae Medical Center from August 1992 to December 2016. All primary breast cancer patients who received treatment at Seoul Metropolitan Government Seoul National University Boramae Medical Center had been registered in the Boramae Hospital Breast Cancer Registry. The number of patients in the registry was 1,456 at the time of this study. We excluded 57 patients who were diagnosed after December 31, 2016. We also excluded 162 patients diagnosed as having stage 0 breast cancer, 39 patients initially diagnosed as having stage IV breast cancer, and 35 patients with no information on their stage. We further excluded 159 patients with no data on MBD, 6 patients diagnosed as having malignant phyllodes tumor, 27 patients who received neoadjuvant chemotherapy, 2 male patients, and 2 patients with age less than 20 or more than 90 years. Finally, we analyzed data of 967 female patients with operable primary invasive breast cancer.

### Clinicopathologic Parameters

We defined patient age as the age at time of diagnosis of primary breast cancer, and described the TNM staging according to the 7^th^ edition of the American Joint Committee on Cancer. Hormone receptor (HRc) status was defined as positive when immunohistochemistry test for either estrogen receptor or progesterone receptor was positive. HRc was defined as negative when both estrogen receptor and progesterone receptor were negative. Human epidermal growth factor receptor 2 (HER2) was defined as negative when immunohistochemistry results were negative or 1+. HER2 was defined as positive when immunohistochemistry results were 3+. When immunohistochemistry results were 2+, positivity of HER2 was defined according to the results of *in situ* hybridization. Histologic grade was defined according to the modified Scarff-Bloom-Richardson grading system. Lymphovascular invasion was defined as positive when either lymphatic invasion or vascular invasion was present. It was defined as negative when both were absent. Body mass index (BMI) was defined as the ratio of body weight (in kilograms) to height (in square meters). All operations with curative intent for breast cancer patients were classified into lumpectomy or mastectomy according to the extent of surgery.

### Mammographic Breast Density

Preoperative MBD was classified according to BI-RADS Atlas 2013 (5^th^ edition)^3^. BI-RADS Atlas for mammography recommends the following four categories of MBD as defined by the visually estimated content of fibroglandular-density tissue within the breast; the breasts are almost entirely fatty (a), there are scattered areas of fibroglandular density (b), the breasts are heterogeneously dense, which may obscure small masses (c), and the breasts are extremely dense, which lowers the sensitivity of mammography (d). In this study, we have described these four categories as fatty, fibroglandular, heterogeneously dense, and extremely dense, respectively. We further defined fatty or fibroglandular as low MBD, and heterogeneously dense or extremely dense as high MBD, respectively. We obtained data for preoperative MBD from the database of the Boramae Hospital Breast Cancer Registry. For this database, MBD data were obtained from the final and formal interpretation report on the preoperative mammography of each subject. In our institute, a mammogram report is made by single radiologist with no consensus. Seven experienced radiologists had been involved in interpretation of mammograms which were utilized in this study at the start time of this study. All radiologists were or are, faculty members of our hospital as professors in the Department of Radiology. All of them graduated from a four year course of resident training in the Department of Radiology and then, finished a two year course of fellowship training for subspecialty in the area of breast cancer. All of them worked or have been working in the Breast Center of our institute as professors in the Department of Radiology. The mean experience period of MBD interpretation is approximately 5 years. All of the mammograms were obtained before pathologic diagnosis of primary breast cancer. Average time between mammography and diagnosis was approximately 2 weeks (median, 13 days; range, 4~89 days).

### Statistical Analyses

Two-sample t-test was used to determine differences in expression levels of biological parameters, while Pearson’s χ^2^ test was used to determine differences in clinicopathologic characteristics between groups. The p for trend value was calculated using the linear by linear association test in a Pearson’s χ^2^ test. We analyzed overall survival and disease-free survival, defining the time intervals for each as the time from operation to death from any cause, and the time from operation to recurrence of any type, respectively. Breast cancer recurrence types included local recurrence, regional recurrence and distant recurrence. Contralateral breast cancer development was not included in breast cancer recurrence types but it was also analyzed in this study. The Kaplan-Meier estimator was used to analyze survival rates and log-rank test was used to determine the significance of differences between two or more survival curves. Cox proportional hazards model was used for multivariable analysis. MBD was adjusted with all 14 factors including 9 clinicopathologic factors (tumor size, nodal positivity, estrogen receptor, progesterone receptor, HER2, histologic grade, lymphovascular invasion, age, and BMI) and 5 treatment factors (operation, radiation therapy, chemotherapy, Herceptin therapy, and endocrine therapy) by multivariate analysis.

Hazard ratio (HR) and 95% confidence interval (CI) were calculated. All statistical analyses were carried out using IBM SPSS Statistics, version 20.0 (IBM Corp., Armonk, NY, USA). All tests were two-tailed. Statistical significance was considered achieved when p value was less than 0.05.

## Results

### Clinicopathologic Characteristics

The total number of subjects was 967 and their mean age was 54.3 ± 12.3 years (median, 52.0 years; range, 25–87 years). Operation dates were between August 1992 and December 2016, and the mean follow-up period for overall survival was 70.8 ± 54.1 months (median, 56.0 months; range, 1~298 months). The total number of deaths and recurrences during this period was 94 (9.7%) and 101 (10.4%), respectively. Among 873 subjects who survived, 652 patients had no evidence of disease, 68 patients had breast cancer recurrence, and 153 patients were missed to follow-up at the reference time of final follow-up (December 2016). The clinicopathologic characteristics of all subjects according to MBD are summarized in Table 1. The proportions of fatty, fibroglandular, heterogeneously dense, and extremely dense breasts were 15.3%, 29.1%, 36.9%, and 18.7%, respectively. Low and high MBD accounted for 44.4% and 55.6% of all subjects, respectively. The mean age of patients with high MBD was significantly lower than that of patients with low MBD. The high MBD group showed a higher proportion of patients with age ≤50 years, BMI ≤ 25 kg/m^2^, and positive HRc compared to the low MBD group. The high MBD group was also associated with a higher proportion of patients who received chemotherapy or radiation therapy.

**Table 1:** Clinicopathologic characteristics of all subjects according to mammographic breast density.

Characteristics	Mammographic Breast Density^a^											Mammographic Breast Density^b^				
	Fatty		Fibroglandular		Heterogeneously dense		Extremely dense		Sum		*p* for Trend	Low		High		*p* ^c^
	No.	%	No.	%	No.	%	No.	%	No.	%		No.	%	No.	%	
All	148	15.3%	281	29.1%	357	36.9%	181	18.7%	967	100.0%		429	44.4%	538	55.6%	
Mean age (years)	66.8 ± 10.0		59.1 ± 10.8		50.2 ± 9.6		44.7 ± 8.8		54.3 ± 12.3			61.7 ± 11.1		48.3 ± 9.7		<0.001
Tumor size (cm)											0.699					0.378
≤2	75	50.7%	126		178	49.9%	90	49.7%	469	48.6%		201	47.0%	268	49.8%	
>2	73	49.3%	154	55.0%	179	50.1%	91	50.3%	497	51.4%		227	53.0%	270	50.2%	
Nodal positivity											0.464					0.818
Negative	108	73.0%	165	58.9%	230	64.4%	117	64.6%	620	64.2%		273	63.8%	347	64.5%	
Positive	40	27.0%	115	41.1%	127	35.6%	64	35.4%	346	35.8%		155	36.2%	191	35.5%	
Stage											0.924					0.477
Stage I	66	44.6%	97	34.6%	142	39.8%	75	41.4%	380	39.3%		163	38.1%	217	40.3%	
Stage II, III	82	55.4%	183	65.4%	215	60.2%	106	58.6%	586	60.7%		265	61.9%	321	59.7%	
Hormonal receptor											0.049					0.024
Negative	39	26.5%	77	28.1%	76	21.7%	37	20.4%	229	24.0%		116	27.6%	113	21.2%	
Positive	108	73.5%	197	71.9%	275	78.3%	144	79.6%	724	76.0%		305	72.4%	419	78.8%	
Estrogen receptor											0.049					0.067
Negative	48	32.7%	87	31.8%	98	27.8%	44	24.3%	277	29.0%		135	32.1%	142	26.6%	
Positive	99	67.3%	187	68.2%	254	72.2%	137	75.7%	677	71.0%		286	67.9%	391	73.4%	
Progesterone receptor											0.011					0.003
Negative	58	39.5%	117	42.7%	115	32.8%	56	30.9%	346	36.3%		175	41.6%	171	32.1%	
Positive	89	60.5%	157	57.3%	236	67.2%	125	69.1%	607	63.7%		246	58.4%	361	67.9%	
HER2											0.054					0.280
Negative	112	81.2%	194	76.4%	252	77.3%	113	70.2%	671	76.3%		306	78.1%	365	74.9%	
Positive	26	18.8%	60	23.6%	74	22.7%	48	29.8%	208	23.7%		86	21.9%	122	25.1%	
Histologic grade											0.330					0.198
1,2	80	58.4%	157	60.2%	219	64.8%	106	61.6%	562	61.9%		237	59.5%	325	63.7%	
3	57	41.6%	104	39.8%	119	35.2%	66	38.4%	346	38.1%		161	40.5%	185	36.3%	
Negative	95	71.4%	162	63.8%	197	62.7%	104	63.8%	558	64.6%		257	66.4%	301	63.1%	
Positive	38	28.6%	92	36.2%	117	37.3%	59	36.2%	306	35.4%		130	33.6%	176	36.9%	
Age (years)											<0.001					<0.001
≤50	9	6.1%	64	22.8%	197	55.2%	144	79.6%	414	42.8%		73	17.0%	341	63.4%	
>50	139	93.9%	217	77.2%	160	44.8%	37	20.4%	553	57.2%		356	83.0%	197	36.6%	
BMI (kg/m^2^)											<0.001					<0.001
≤25	68	45.9%	131	46.6%	238	66.9%	159	87.8%	596	61.7%		199	46.4%	397	73.9%	
>25	80	54.1%	150	53.4%	118	33.1%	22	12.2%	370	38.3%		230	53.6%	140	26.1%	
Operation											0.063					0.062
Lumpectomy	62	41.9%	103	36.7%	150	42.0%	89	49.2%	404	41.8%		165	38.5%	239	44.4%	
Mastectomy	86	58.1%	178	63.3%	207	58.0%	92	50.8%	563	58.2%		264	61.5%	299	55.6%	
Radiation therapy											0.008					0.028
No	86	58.1%	151	53.7%	179	50.1%	80	44.2%	496	51.3%		237	55.2%	259	48.1%	
Yes	62	41.9%	130	46.3%	178	49.9%	101	55.8%	471	48.7%		192	44.8%	279	51.9%	
Chemotherapy											<0.001					<0.001
No	69	46.6%	80	28.5%	84	23.5%	41	22.7%	274	28.3%		149	34.7%	125	23.2%	
Yes	79	53.4%	201	71.5%	273	76.5%	140	77.3%	693	71.7%		280	65.3%	413	76.8%	
Herceptin therapy											0.952					0.986
No	134	90.5%	251	89.3%	320	89.6%	163	90.1%	868	89.8%		385	89.7%	483	89.8%	
Yes	14	9.5%	30	10.7%	37	10.4%	18	9.9%	99	10.2%		44	10.3%	55	10.2%	
Endocrine therapy											0.175					0.072
No	34	23.0%	90	32.0%	91	25.5%	37	20.4%	252	26.1%		124	28.9%	128	23.8%	
Yes	114	77.0%	191	68.0%	266	74.5%	144	79.6%	715	73.9%		305	71.1%	410	76.2%	

Abbreviations: BMI, body mass index; HER2; human epidermal growth factor receptor 2.

^a^Mammographic breast density was classified to 4 categories as fatty, fibroglandular, heterogeneously dense, and extremely dense according to BI-RADS Atlas 2013 (5th edition).

^b^Fatty and fibroglandular categories were defined as low mammographic breast density. Heterogeneously dense and extremely dense categories were defined as high mammographic breast density.

^c^*P* value for mean age was calculated by *t*-test and all the other *p* values were calculated by *χ*^*2*^ test.

### Survival Analysis

**Figure 1:**
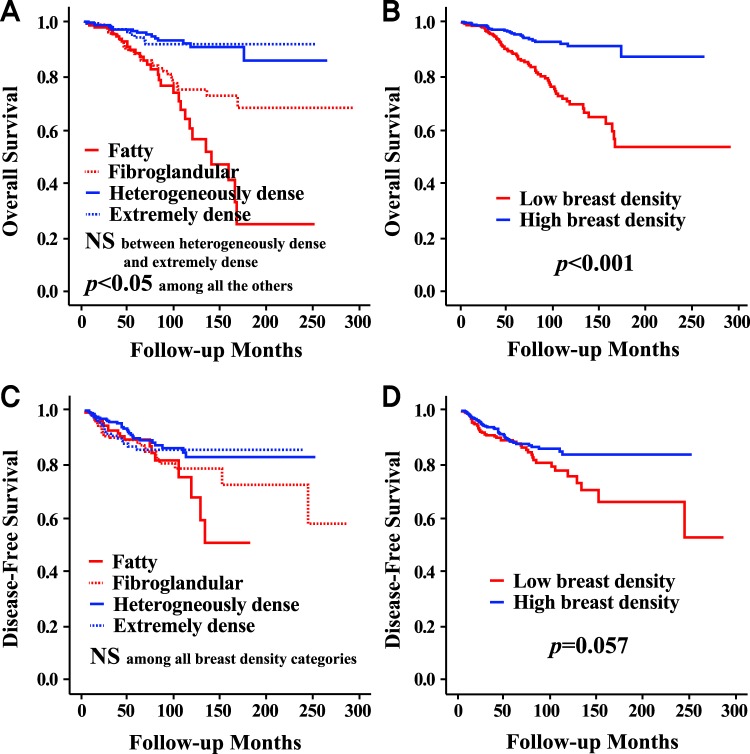
Survival curves according to mammographic breast density in all subjects. Overall survival curves according to 4 categories of mammographic breast density (**A**) and according to high or low mammographic breast density (**B**). Disease-free survival curves according to 4 categories of mammographic breast density (**C**) and according to high or low mammographic breast density (**D**). Abbreviations: NS, not significant.

**Figure 2:**
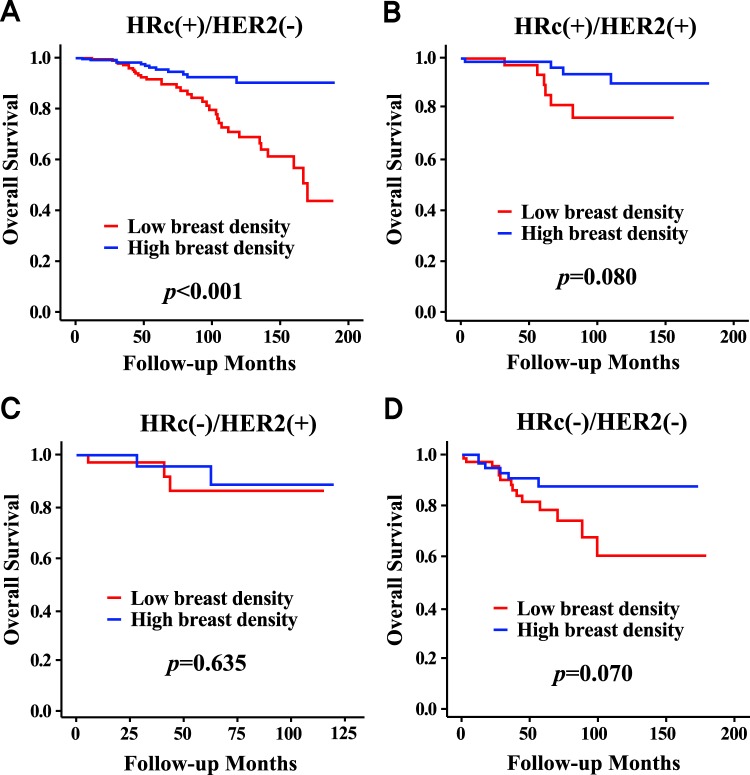
Overall survival curves according to mammographic breast density in patients with each breast cancer subtype. HRc(+)/HER2(−) (**A**), HRc(+)/HER2(+) (**B**), HRc(−)/HER2(+) (**C**), and HRc(−)/HER2(−) (**D**). Abbreviations: HER2, human epidermal growth factor receptor 2; HRc, hormonal receptor.

The high MBD group showed a superior overall survival rate compared to the low MBD group (p < 0.001), but there was no significant difference between the two groups in terms of the disease-free survival rate (Fig. 1). When MBD was classified into four categories, the p values by log-rank test were significant among all categories, except between heterogeneously dense, and extremely dense, with respect to overall survival. The p values were not significant among any breast density categories with respect to disease-free survival. The high MBD group showed a higher overall survival rate compared to the low MBD group only in the HRc(+)/HER2(−) subgroup (p < 0.001), and there were no survival differences between the two groups in the other subgroups of HRc(+)/HER2(+), HRc(−)/HER2(+), and HRc(−)/HER2(−) (Fig. 2). There were no differences between the high and low MBD groups in terms of local recurrence or distant recurrence (Fig. S1). The low MBD group showed a higher regional recurrence rate compared to the high MBD group (p = 0.013), but the contralateral breast cancer development rate was higher in the high MBD group (p = 0.034). Detailed regional recurrence rates and incidence rates of contralateral breast cancer according to mammographic breast density are described in Table S3.

### Subgroup Analysis

Subgroup analyses with a forest plot revealed that prognoses in the high MBD group were better than those in the low MBD group in subgroups, regardless of tumor size, nodal positivity, stage, estrogen receptor, progesterone receptor, histologic grade, lymphovascular invasion, BMI, operation, radiation therapy, and chemotherapy (Table 2). Although the high MBD group showed superior prognosis in the subgroup with negative HER2 and in patients who received endocrine therapy, there were no differences in the subgroup with positive HER2 and in patients who did not receive endocrine therapy. Notably, the high MBD group showed better prognosis in the subgroup aged >50 years, but no difference was observed in the subgroup aged ≤50 years. In all subjects, the high MBD group showed superior prognosis compared to the low MBD group (HR, 0.282; 95% CI, 0.179–0.443).

**Table 2:** Subgroup analysis of hazard ratios according to mammographic breast density regarding overall survival in all subjects.

Characteristics		Subject Number	Low MBD	High MBD	HR^a^	CI (95%)		*p*	Forest Plot^b^
Total		907	414	493	0.282	0.179	0.443	<0.001	
Tumor size (cm)	≤2	440	194	246	0.214	0.085	0.536	0.001	
	>2	466	219	247	0.330	0.196	0.556	<0.001	
Nodal positivity	Negative	579	264	315	0.217	0.107	0.440	<0.001	
	Positive	327	149	178	0.357	0.197	0.645	0.001	
Stage	Stage I	353	157	196	0.178	0.050	0.632	0.008	
	Stage II, III	553	256	297	0.319	0.196	0.519	<0.001	
Hormonal receptor	Negative	215	113	102	0.363	0.160	0.820	0.015	
	Positive	681	295	386	0.256	0.149	0.442	<0.001	
Estrogen receptor	Negative	262	131	131	0.428	0.212	0.861	0.017	
	Positive	635	277	358	0.218	0.120	0.398	<0.001	
Progesterone receptor	Negative	327	170	157	0.317	0.159	0.629	0.001	
	Positive	569	238	331	0.257	0.141	0.471	<0.001	
HER2	Negative	638	298	340	0.312	0.182	0.537	<0.001	
	Positive	190	82	108	0.410	0.146	1.157	0.092	
Histologic grade	1,2	529	230	299	0.204	0.103	0.405	<0.001	
	3	321	155	166	0.333	0.174	0.637	0.001	
Lymphovascular invasion	Negative	527	251	276	0.161	0.062	0.420	<0.001	
	Positive	285	122	163	0.315	0.172	0.577	<0.001	
Age (years)	≤50	379	69	310	0.495	0.220	1.118	0.091	
	>50	528	345	183	0.254	0.126	0.513	<0.001	
BMI (kg/m^2^)	≤25	550	189	361	0.213	0.118	0.384	<0.001	
	>25	356	225	131	0.430	0.211	0.877	0.020	
Operation	Lumpectomy	383	160	223	0.148	0.032	0.686	0.015	
	Mastectomy	524	254	270	0.325	0.202	0.522	<0.001	
Radiation therapy	No	468	230	238	0.347	0.203	0.591	<0.001	
	Yes	439	184	255	0.188	0.080	0.445	<0.001	
Chemotherapy	No	260	144	116	0.185	0.072	0.474	<0.001	
	Yes	647	270	377	0.382	0.221	0.658	0.001	
Endocrine therapy	No	237	120	117	0.485	0.232	1.013	0.054	
	Yes	670	294	376	0.221	0.124	0.396	<0.001	
		

Abbreviations: BMI, body mass index; CI, confidence interval; HER2, human epidermal growth factor receptor 2; HR, hazard ratio.

^a^HRs are the relative risks of the high mammographic breast density group with reference of the low mammographic breast density group regarding overall survival by Cox proportional hazards model.

^b^In the forest plot, a HR value of less than 1 favors the high mammographic breast density group against the low mammographic breast density group regarding overall survival. The red circles mean statistical significance and the blue squares mean no statistical significance. The green diamond means the result of total subjects.

### Multivariable Analysis

MBD was a significant independent prognostic factor (HR, 0.382; 95% CI, 0.206–0.708) after being adjusted with 14 factors including nine clinicopathologic factors and five treatment factors (Table 3).

**Table 3:** Multivariable analysis in all subjects regarding mammographic breast density in terms of overall survival.

Characteristics	HR	95% CI		*p*
Mammographic breast density, high vs low	0.382	0.206	0.708	0.002
Tumor size (cm), >2 vs ≤2	2.132	1.084	4.194	0.028
Nodal positivity, positive vs negative	1.103	0.605	2.013	0.749
Estrogen receptor, positive vs negative	0.757	0.391	1.469	0.411
Progesterone receptor, positive vs negative	0.612	0.313	1.196	0.151
HER2, positive vs negative	0.978	0.507	1.885	0.946
Histologic grade, 3 vs 1,2	1.406	0.793	2.492	0.243
Lymphovascular invasion, positive vs negative	2.525	1.315	4.851	0.005
Age (years), >50 vs ≤50	1.150	0.599	2.210	0.674
BMI (kg/m^2^), >25 vs ≤25	0.826	0.493	1.384	0.468
Operation, mastectomy vs lumpectomy	2.566	1.152	5.715	0.021
Radiation therapy, yes vs no	1.096	0.574	2.092	0.782
Chemotherapy, yes vs no	0.250	0.140	0.444	<0.001
Herceptin therapy, yes vs no	0.438	0.093	2.064	0.297
Endocrine therapy, yes vs no	1.227	0.563	2.673	0.607

Abbreviations: BMI, body mass index; CI, confidence interval; HR, hazard ratio.

### Mammographic Breast Density in Patients with Age >50 Years

**Figure 3:**
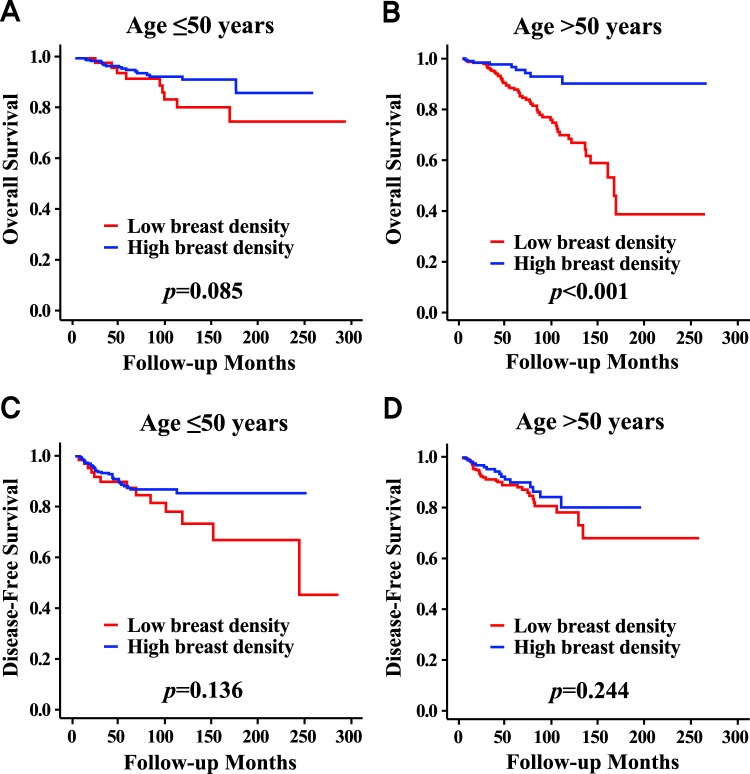
Survival curves according to mammographic breast density in patients with age ≤50 years and in patients with age >50 years. Overall survival curves according to mammographic breast density in patients with ≤50 years (**A**) and in patients with >50 years (**B**). Disease-free survival curves according to mammographic breast density in patients with ≤50 years (**C**) and in patients with >50 years (**D**).

The high MBD group showed a higher overall survival than the low MBD group only in the subgroup aged >50 years (p < 0.001), while a survival difference was not observed between the two groups in the subgroup aged ≤50 years (Fig. 3). There were no survival differences between the two groups regardless of age subgroups in terms of disease-free survival. In the subgroup aged >50 years, the mean age of patients in the high MBD group was significantly lower than that of patients in the low MBD group (Table S1). The high MBD group showed a higher proportion of patients with BMI ≤ 25 kg/m^2^. In the subgroup aged >50 years, MBD was a significant independent prognostic factor by multivariable analysis (HR, 0.399; 95% CI, 0.181–0.881; Table S2).

### Mammographic Breast Density and Age

In all subjects, MBD was closely related to age; as higher MBD was associated with lower mean age in terms of the four categories (two-sample t-test; all p < 0.001; Fig. S2A) and the two MBD groups (two-sample t-test; p < 0.001; Fig. S2B). Although the relationship between MBD and mean age was weak in the subgroup aged ≤50 years, it was strong in the subgroup aged >50 years (Fig. S2).

## Discussion

In this study, we have shown that high MBD is associated with a superior prognosis compared to low MBD, and that MBD is a strong independent prognostic factor in operable primary invasive female breast cancer, especially in patients older than 50 years and with the HRc(+)/HER2(−) subtype. Among prior studies reporting on results regarding the relationship between MBD and breast cancer, most studies focused on the role of MBD as an etiologic factor. Only a limited number of published studies have reported on the role of MBD as a prognostic factor, but the results were inconsistent and controversial. Some papers have reported adverse effects of low MBD on breast cancer prognosis with results similar to the findings of this study. One study reported that patients with low MBD (<25%) showed worse prognosis compared to those with mixed breast density (>25%), and patients with very low MBD (<10%) showed the strongest significance compared to the remaining patients (HR, 3.275; 95% CI, 1.750–6.127)^10^. The authors also reported that patients with very low MBD (<10%) had a clinically worse disease-free survival compared to those with mixed MBD (>10%) by multivariable analysis (HR, 2.790; 95% CI, 1.724–4.516)^11^. Other papers reported the favorable prognostic effect of low MBD on breast cancer. One study reported that median progression-free survival in patients with low breast density (18.4 months; 95% CI, 14.9–22.2) was significantly better than that in patients with high breast density (9.3 months, 95% CI 8.5–13.6) with a p value of 0.002^12^. Another study reported that dense breast was significantly associated with an increased breast cancer-specific mortality (HR, 1.91; 95% CI, 1.26–2.91) compared to nondense breast^13^. However, another paper reported that there was no association between MBD and breast cancer prognosis. A study using data of Breast Cancer Surveillance Consortium (BCSC) reported that high MBD (BI-RADS 4) was not associated with breast cancer-specific survival (HR, 0.92; 95% CI, 0.71–1.19) or overall survival (HR, 0.83; 95% CI, 0.68–1.02) compared to low MBD (BI-RADS 2) by multivariable analysis^14^. A further study reported that MBD was not associated with breast cancer-specific survival (HR, 0.95; 95% CI, 0.79–1.15) or overall survival (HR, 1.08; 95% CI, 0.98–1.20)^15^. Eriksson *et al*. also reported that MBD was not associated with breast cancer-specific survival or overall survival in postmenopausal breast cancer patients^16^. However, the results of our study strongly support a favorable prognostic role of high MBD in terms of overall survival in breast cancer compared to low MBD.

The proposed mechanisms underlying the prognostic value of MBD could be explained by three factors, including clinicopathologic factors, treatment factors, and other factors. First, the high MBD group was associated with favorable factors such as younger age, lower BMI, and a higher proportion of patients with positive HRc or progesterone receptor. Second, the high MBD group could be associated with more active treatments. Although there were no differences in terms of tumor size, nodal positivity, and stage between the high MBD group and the low MBD group, the proportion of patients receiving adjuvant chemotherapy was higher in the high MBD group. Third, other factors could be associated with these results. After adjusting MBD with both clinicopathologic and treatment factors, MBD still remained a significant independent prognostic factor by multivariable analysis in terms of overall survival. This implies that factors other than clinicopathologic and treatment, could be significant effectors explaining this relationship. Socioeconomic status, exercise, dietary habit, health insurance, life style, for example, could be relevant contributors, but further studies would be needed in order to validate roles for these.

As all of the subjects in this study were Asian, racial differences should be considered. According to data from 3,865,070 screening mammography examinations interpreted by radiologists who participated in the BCSC, the proportions of fatty, fibroglandular, heterogeneously dense, and extremely dense breasts were about 10%, 40%, 40%, and 10%, respectively^3^. The majority of the study population in the BCSC were white (http://www.bcsc-research.org/). In our study, the proportions of fatty, fibroglandular, heterogeneously dense, and extremely dense breasts were approximately 15%, 30%, 35%, and 20%, respectively. BCSC data showed that high and low MBD accounted for 50% and 50%, respectively, but our data indicated that the proportions were approximately 45% and 55%, respectively. Asian women have been reported to have more dense breasts compared to white women. A previous study reported that the proportions of patients having extremely dense breasts were 27.1% and 12.5% in Asian and White women respectively, using data of 15,292 women without a history of breast cancer^17^. Another study also reported that HR for having dense breasts versus fatty breasts, comparing Asian to white women was 1.3 (95% CI, 1.2–1.5) after full adjustment using data of 28,501 women without a history of breast cancer^18^.

Although there is considerable variation, MBD changes inversely with age; younger women tend to have denser breasts compared to older women. A previous study reported that there was a significant inverse relationship between age and MBD (p < 0.001), and proportions of dense breast were 74%, 57%, 44%, and 36% for women in their 40 s, 50 s, 60 s, and 70 s, respectively^19^. Age is also closely related to breast cancer prognosis. Chen *et al*. analyzed 133,057 female breast cancer patients from 2004 to 2008 using the Surveillance, Epidemiology, and End Results database^20^. In our study, high MBD was strongly associated with younger age. The strong association between MBD and the age factor could be one of the major causalities behind better prognosis with high MBD.

Obesity has been reported to be a poor prognostic factor in breast cancer. Obesity increases the risk of breast cancer recurrence and death by approximately 35–40% and this is most clearly established for estrogen receptor-positive breast cancer^21^. A previous study reported that high BMI was an independent prognostic factor in breast cancer for worse outcomes with respect to overall survival (p = 0.03)^22^. Chan *et al*. reported the most recent and extensive meta-analysis results using 213,075 patients from 82 studies. They reported that the summary relative risks for total mortality and breast cancer–specific mortality for obese versus normal-weight patients at baseline were 1.41 (95% CI, 1.29–1.53) and 1.35 (95% CI, 1.24–1.47), respectively^23^. In this study, low MBD was strongly associated with high BMI and it could partly explain the worse prognosis found in the low MBD group.

In this study, high MBD showed higher overall survival rates in all subjects, but this was found only in patients older than 50 years on subgroup analyses. This finding could be one of the main reasons for the prognostic effect of MBD only in patients with age >50 years in this study. Multivariable analysis revealed similar findings, showing that MBD was a significant independent prognostic factor in both unselected breast cancer patients and breast cancer patients with age >50 years, but not in patients with age ≤50 years. According to breast cancer subtypes, MBD was a significant prognostic factor only in the HRc(+)/HER2(−) subtype. Subgroup analyses revealed that the prognostic impact of MBD was more prominent in the subgroup with positive estrogen receptor, positive progesterone receptor, or negative HER2 compared to negative estrogen receptor, negative progesterone receptor, or positive HER2, respectively. The prognostic influence of MBD was different across subtypes of breast cancer, and it was observed only in the HRc(+)/HER2(−) subtype.

Low MBD was significantly associated with higher regional recurrence and lower contralateral breast cancer development, but was not associated with local recurrence or distant recurrence. As a whole, MBD was not a significant factor with respect to disease-free survival, and breast cancer recurrence and therefore MBD is considered to be a minor prognostic factor in our study. Previous papers have reported that high MBD was closely associated with increased locoregional recurrence, but not with distant recurrence^24–26^. Eriksson *et al*. reported that in postmenopausal patients, breast cancer originating in dense breasts (≥25%) was associated with higher local recurrence (HR, 1.92; p = 0.039) and locoregional recurrence (HR, 1.67; p = 0.033) compared to patients with less dense breasts (25% density), but it was not associated with distant recurrence^16^. A previous study reported that dense breast was associated with an increased risk of contralateral breast cancer development (HR, 1.80; 95% CI, 1.22–2.64) compared to nondense breast^27^.

This study has limitations. First, this was a retrospective cohort study including a relatively small number of subjects, and therefore limited statistical power particularly for subgroup analysis. Second, the main concern of this study was the prognostic influence of preoperative mammographic breast density in operable invasive female breast cancer and we selected the subjects accordingly. Selection bias could be a limitation of this study which can occur as a result of excluding relatively large numbers of subjects. We excluded patients with stage 0 or stage IV as initial diagnosis, patients who received neoadjuvant chemotherapy, patients diagnosed as malignant phyllodes tumor, male patients, patients less than 20 or more than 90 years old, and patients with insufficient information for this study. Third, MBD data has limitations. All data for MBD were obtained from the Boramae Hospital Breast Cancer Registry database based on the mammogram report which was made by single radiologist with no consensus. Although all radiologists who had been involved in interpretation of MBD were, or are, faculty members of our hospital as professors in the Department of Radiology specializing in breast cancer, MBD data were not reviewed independently by radiologist(s) in a controlled manner. Interpretation of MBD in this study was based on visual qualitative analysis which is considered less accurate than quantitative or automated measurements. There was no adjustment between analog mammography films and digital mammography. Fourth, the results of breast cancer-specific survival were not presented in this study, as data for this analysis were not available. Fifth, the change in the prognostic effect of MBD according to different time points was not evaluated in this study and further study is needed to investigate this parameter. Sixth, the recruitment period is relatively long, and there could be biases associated with this variable. There have been chronologic changes in basic clinicopathologic features, diagnostic methods, treatment modalities, and other factors associated with prognosis. Lastly, as this study was based on the Boramae Hospital Breast Cancer Registry database which had limited clinicopathologic information, some important factors could not be analyzed directly. For example, as the registry had no information regarding menopausal status, we analyzed age subgroups with arbitrary cut-off value of 50 years which surrogates for the menopausal status of the subjects. Additionally, although MBD was adjusted with 14 factors including 9 clinicopathologic factors and 5 treatment factors, there could be still other possible confounding factors which were not analyzed in this study.

In conclusion, the high MBD group was associated with superior overall survival rates. Preoperative MBD was a strong independent prognostic factor in operable primary invasive female breast cancer, especially in patients with age >50 years and the HRc(+)/HER2(−) subtype. Favorable clinicopathologic features, active treatments, and other factors could contribute to this causality. Large scale, well designed studies are needed in the near future to validate the relationship between MBD and breast cancer prognosis.

### Ethical Statement

The institutional review boards approved this study (Seoul Metropolitan Government Seoul National University Boramae Medical Center, 16-2017-70) and performed in accordance with the principles of the Declaration of Helsinki. The informed consent of this study was waived.

## Electronic supplementary material

Supplementary information
