# Supplementary material for: Prognostic Influence of Preoperative Mammographic Breast Density in Operable Invasive Female Breast Cancer
Source: Sci Rep. 2018 Oct 30;8:16075. doi: 10.1038/s41598-018-34297-8 (PMC6207781; doi:10.1038/s41598-018-34297-8)
Supplement: Supplementary file 1 — Supplementary information [file 41598_2018_34297_MOESM1_ESM.pdf]

Prognostic Influence of Preoperative Mammographic Breast Density in Operable Invasive Female Breast Cancer

Ki-Tae Hwang[1,*,+], A Jung Chu[2,+], Jongjin Kim[1], Jong Yoon Lee[2], Ji Hyun Chang[3], Sohee Oh[4], Young A Kim[5], Jiwoong Jung[6] & Bumjo Oh[7]

[1]Department of Surgery, Seoul Metropolitan Government Seoul National University Boramae Medical Center, Seoul, Republic of Korea
[2]Department of Radiology, Seoul Metropolitan Government Seoul National University Boramae Medical Center, Seoul, Republic of Korea
[3]Department of Radiation Oncology, Seoul Metropolitan Government Seoul National University Boramae Medical Center, Seoul, Republic of Korea
[4]Department of Biostatistics, Seoul Metropolitan Government Seoul National University Boramae Medical Center, Seoul, Republic of Korea
[5]Department of Pathology, Seoul Metropolitan Government Seoul National University Boramae Medical Center, Seoul, Republic of Korea
[6]Department of Surgery, Seoul Medical Center, Seoul, Republic of Korea
[7]Department of Family Medicine, Seoul Metropolitan Government Seoul National University Boramae Medical Center, Seoul, Republic of Korea

[+] **Ki-Tae Hwang and A Jung Chu contributed equally to this work.**

Table S1. Clinicopathologic characteristics according to mammographic breast density in patients with age ≤50 years and in patients with age >50 years.

Characteristics	Mammographic Breast Density (Age ≤50 Years)							Mammographic Breast Density (Age >50 Years)						
	Low		High		Sum		*p* [a]	Low		High		Sum		*p* [a]
	No.	%	No.	%	No.	%		No.	%	No.	%	No.	%	
All	73	17.6%	341	82.4%	414	100.0%		356	64.4%	197	35.6%	553	100.0%	
Mean age (years)	44.2±5.0		42.6±5.5		42.9±5.4		0.017	65.3±8.3		58.2±7.0		62.8±8.5		<0.001
Tumor size (cm)							0.697							0.064
≤2	32	43.8%	158	46.3%	190	45.9%		169	47.6%	110	55.8%	279	50.5%	
>2	41	56.2%	183	53.7%	224	54.1%		186	52.4%	87	44.2%	273	49.5%	
Nodal positivity							0.024							0.704
Negative	36	50.0%	219	64.2%	255	61.7%		237	66.6%	128	65.0%	365	66.0%	
Positive	36	50.0%	122	35.8%	158	38.3%		119	33.4%	69	35.0%	188	34.0%	
Stage							0.157							0.237
Stage I	21	28.8%	128	37.5%	149	36.0%		142	40.0%	89	45.2%	231	41.8%	
Stage II, III	52	71.2%	213	62.5%	265	64.0%		213	60.0%	108	54.8%	321	58.2%	
Hormonal receptor							0.322							0.822
Negative	15	22.7%	59	17.6%	74	18.4%		101	28.5%	54	27.6%	155	28.1%	
Positive	51	77.3%	277	82.4%	328	81.6%		254	71.5%	142	72.4%	396	71.9%	
Estrogen receptor							0.039							0.698
Negative	23	34.8%	77	22.8%	100	24.8%		112	31.5%	65	33.2%	177	32.1%	
Positive	43	65.2%	260	77.2%	303	75.2%		243	68.5%	131	66.8%	374	67.9%	
Progesterone receptor							0.136							0.993
Negative	23	34.8%	87	25.9%	110	27.4%		152	42.8%	84	42.9%	236	42.8%	
Positive	43	65.2%	249	74.1%	292	72.6%		203	57.2%	112	57.1%	315	57.2%	
HER2							0.290							0.430
Negative	49	81.7%	232	75.3%	281	76.4%		257	77.4%	133	74.3%	390	76.3%	
Positive	11	18.3%	76	24.7%	87	23.6%		75	22.6%	46	25.7%	121	23.7%	
Histologic grade							0.592							0.467
1,2	39	60.9%	205	64.5%	244	63.9%		198	59.3%	120	62.5%	318	60.5%	
3	25	39.1%	113	35.5%	138	36.1%		136	40.7%	72	37.5%	208	39.5%	
Lymphovascular invasion							0.178							0.504
Negative	30	51.7%	180	61.2%	210	59.7%		227	69.0%	121	66.1%	348	68.0%	
Positive	28	48.3%	114	38.8%	142	40.3%		102	31.0%	62	33.9%	164	32.0%	
BMI (kg/m^2)							<0.001							<0.001
≤25	35	47.9%	266	78.0%	301	72.7%		164	46.1%	131	66.8%	295	53.4%	
>25	38	52.1%	75	22.0%	113	27.3%		192	53.9%	65	33.2%	257	46.6%	
Operation							0.674							0.205
Lumpectomy	31	42.5%	154	45.2%	185	44.7%		134	37.6%	85	43.1%	219	39.6%	
Mastectomy	42	57.5%	187	54.8%	229	55.3%		222	62.4%	112	56.9%	334	60.4%	
Radiation therapy							0.356							0.979
No	36	49.3%	148	43.4%	184	44.4%		201	56.5%	111	56.3%	312	56.4%	
Yes	37	50.7%	193	56.6%	230	55.6%		155	43.5%	86	43.7%	241	43.6%	
Chemotherapy							0.982							0.079
No	14	19.2%	65	19.1%	79	19.1%		135	37.9%	60	30.5%	195	35.3%	
Yes	59	80.8%	276	80.9%	335	80.9%		221	62.1%	137	69.5%	358	64.7%	
Herceptin therapy							0.504							0.566
No	67	91.8%	304	89.1%	371	89.6%		318	89.3%	179	90.9%	497	89.9%	
Yes	6	8.2%	37	10.9%	43	10.4%		38	10.7%	18	9.1%	56	10.1%	
Endocrine therapy							0.026							0.521
No	23	31.5%	67	19.6%	90	21.7%		101	28.4%	61	31.0%	162	29.3%	
Yes	50	68.5%	274	80.4%	324	78.3%		255	71.6%	136	69.0%	391	70.7%	

Abbreviations: BMI, body mass index; HER2, human epidermal growth factor receptor 2.

[a]*P* value for mean age was calculated by *t*-test and all the other *p* values were calculated by $\chi 2$ test.

Table S2. Multivariable analysis in patients with age >50 years in terms of overall survival.

Characteristics (Age>50)	HR	95% CI		*p*
Mammographic breast density, high vs low	0.399	0.181	0.881	0.023
Tumor size (cm), >2 vs ≤2	2.226	1.016	4.876	0.045
Nodal positivity, positive vs negative	1.413	0.630	3.171	0.402
Estrogen receptor, positive vs negative	0.476	0.190	1.192	0.113
Progesterone receptor, positive vs negative	1.417	0.539	3.725	0.480
HER2, positive vs negative	1.162	0.554	2.434	0.691
Histologic grade, 3 vs 1,2	1.491	0.723	3.077	0.279
Lymphovascular invasion, positive vs negative	1.641	0.723	3.724	0.237
BMI (kg/m^2), >25 vs ≤25	0.984	0.541	1.788	0.957
Operation, mastectomy vs lumpectomy	2.994	1.059	8.466	0.039
Radiation therapy, yes vs no	1.942	0.847	4.451	0.117
Chemotherapy, yes vs no	0.136	0.067	0.276	<0.001
Herceptin therapy, yes vs no	0.322	0.038	2.742	0.300
Endocrine therapy, yes vs no	0.943	0.302	2.944	0.919

Abbreviations: BMI, body mass index; CI, confidence interval; HR, hazard ratio.

Table S3. Detailed regional recurrence rates and incidence rates of contralateral breast cancer according to mammographic breast density.

Follow-up Months	Regional Recurrence						Contralateral Breast Cancer					
	Low Breast Density			High Breast Density			Low Breast Density			High Breast Density		
	Subject No	Event No[a]	Rate	Subject No	Event No[a]	Rate	Subject No	Event No[a]	Rate	Subject No	Event No[a]	Rate
60 months	425	14	4.0%	535	10	2.8%	425	1	0.2%	535	8	2.6%
120 months	146	20	10.1%	200	11	3.9%	148	2	1.4%	197	11	5.3%
180 months	36	20	10.1%	56	11	3.9%	38	2	1.4%	56	12	8.2%
240 months	9	20	10.1%	8	11	3.9%	9	2	1.4%	7	12	8.2%

[a]Cumulative number of events.

Figure S1. Curves for breast cancer recurrence and contralateral breast cancer development according to mammographic breast density in all subjects. Local recurrence (A), regional recurrence (B), distant recurrence (C), and contralateral breast cancer development (D).

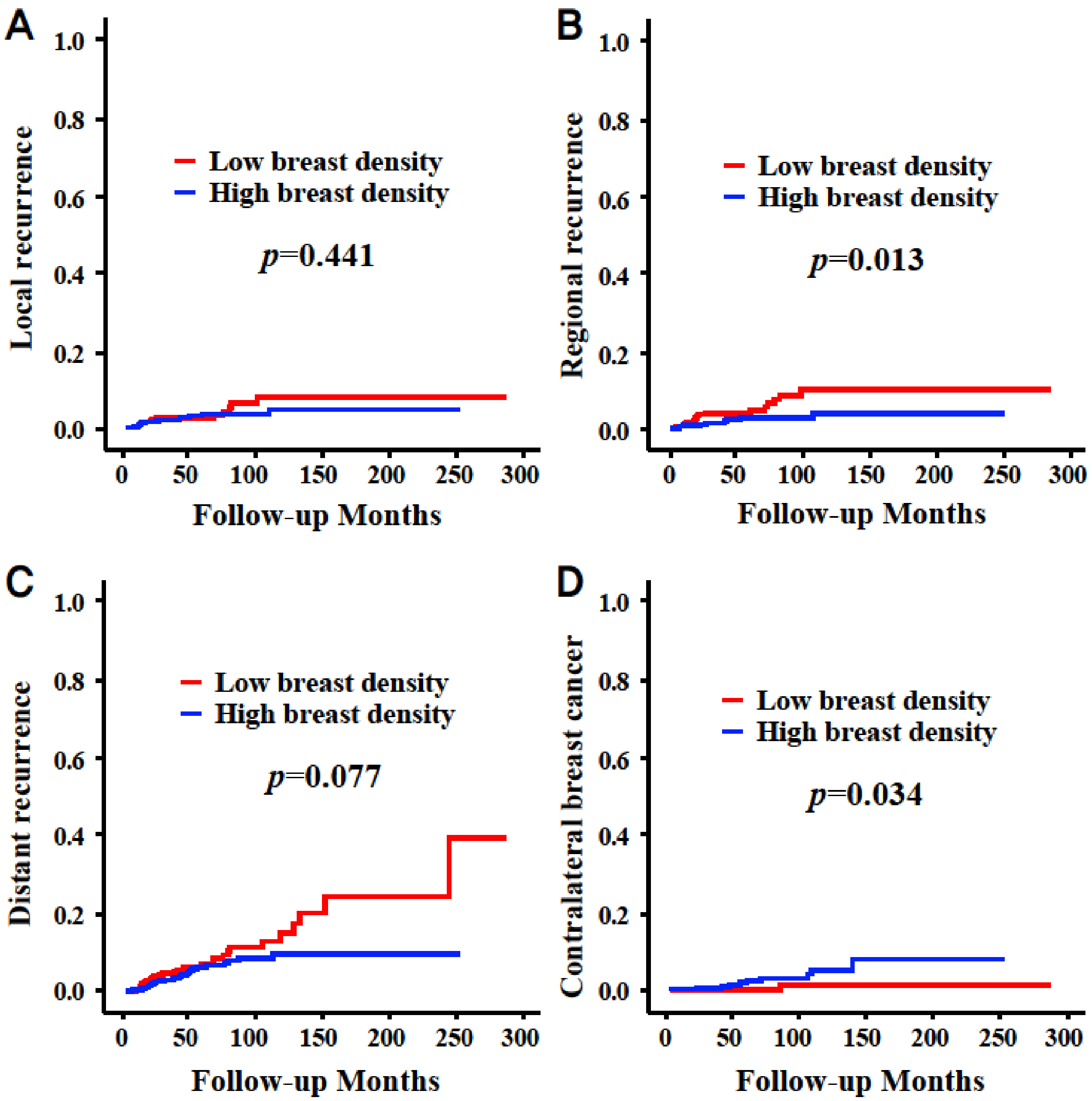

Figure S2. Box plots showing the relationships between mammographic breast density and age. Box plots for 4 categories of mammographic breast density in all subjects (A), in patients with age ≤50 years (C), and in patients with age >50 years (E). Box plots for high or low mammographic breast density in all subjects (B), in patients with age ≤50 years (D), and in patients with age >50 years (F).

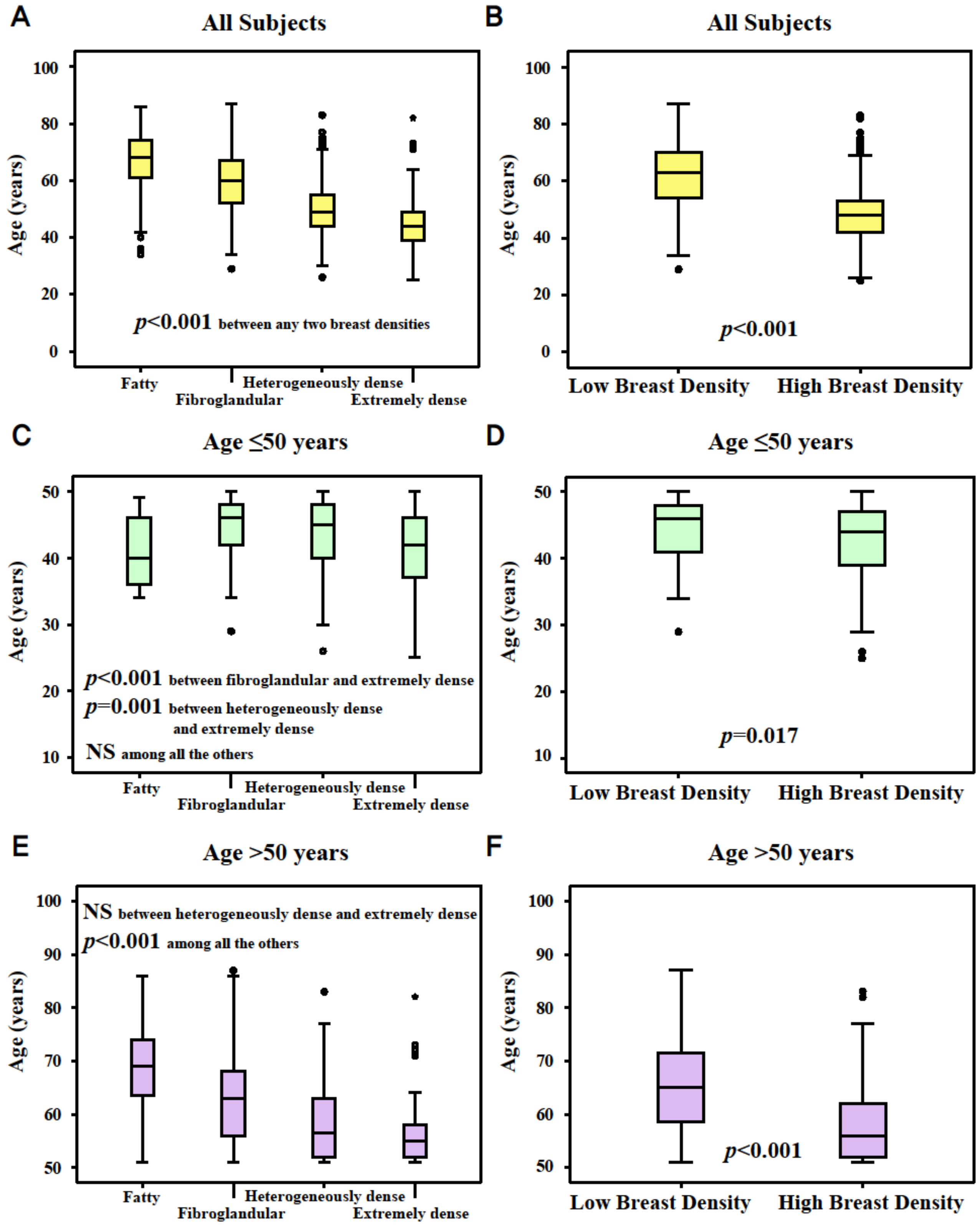

Abbreviations: NS, not significant.
